# Metabotropic Glutamate Receptor Subtype 5 Positron-Emission-Tomography Radioligands as a Tool for Central Nervous System Drug Development: Between Progress and Setbacks

**DOI:** 10.3390/ph16081127

**Published:** 2023-08-10

**Authors:** Anne-Claire Dupont, Nicolas Arlicot, Johnny Vercouillie, Sophie Serrière, Serge Maia, Frédérique Bonnet-Brilhault, Maria-Joao Santiago-Ribeiro

**Affiliations:** 1 Radiopharmacie, CHRU de Tours, 37000 Tours, France; 2 UMR 1253, iBrain, Tours University, INSERM, 37000 Tours, France; 3 CIC 1415, Tours University, INSERM, 37000 Tours, France; 4 Excellence Center for Autism and Neurodevelopmental Disorders, CHRU de Tours, 37000 Tours, France; 5 Nuclear Medicine Department, CHRU de Tours, 37000 Tours, France

**Keywords:** mGluR5, PET, biomarker, drug development, neuroimaging

## Abstract

The metabotropic glutamate receptor subtype 5 (mGluR5) is a class C G-protein-coupled receptor (GPCR) that has been implicated in various neuronal processes and, consequently, in several neuropsychiatric or neurodevelopmental disorders. Over the past few decades, mGluR5 has become a major focus for pharmaceutical companies, as an attractive target for drug development, particularly through the therapeutic potential of its modulators. In particular, allosteric binding sites have been targeted for better specificity and efficacy. In this context, Positron Emission Tomography (PET) appears as a useful tool for making decisions along a drug candidate’s development process, saving time and money. Thus, PET provides quantitative information about a potential drug candidate and its target at the molecular level. However, in this area, particular attention has to be given to the interpretation of the PET signal and its conclusions. Indeed, the complex pharmacology of both mGluR5 and radioligands, allosterism, the influence of endogenous glutamate and the choice of pharmacokinetic model are all factors that may influence the PET signal. This review focuses on mGluR5 PET radioligands used at several stages of central nervous system drug development, highlighting advances and setbacks related to the complex pharmacology of these radiotracers.

## 1. Introduction

### 1.1. mGluR5 as a Target of Interest

Glutamate, the main excitatory neurotransmitter in the central nervous system (CNS), plays a major role in brain function, from the early stages of neurogenesis to the brain aging process [1]. Glutamatergic circuits are largely involved in different aspects of CNS development (cell migration, synaptogenesis) but also participate in major neural functions of the brain such as learning, memory, cognition, movement or nociception [2]. Nevertheless, if found in excessive amounts, glutamate could lead to neuronal dysfunction and become associated with various neurological and psychiatric disorders. The function of glutamate, whether physio- or pathological, is conditioned by its concentration in the extracellular medium. The glutamate receptors are divided into two main classes: ionotropic receptors (AMPA, kainate and NMDA receptors) that are responsible for rapid excitatory effects [3], and metabotropic receptors (mGluR) that are involved in modulating glutamatergic neurotransmission. The metabotropic glutamate receptors are categorized into three distinct subtypes, based on their structure and functions: group I, comprising mGluR1 and mGluR5; group II, comprising mGluR2 and mGluR3; and group III, comprising mGluR4 and mGluR6-8. The mGluR belongs to the G-protein-coupled receptors (GPCRs), which represent the largest class of drug targets, accounting for over 40% of marketed drugs [4,5]. Among these receptors, mGluR5 has been shown to play critical roles in synaptic plasticity and neuronal development [6,7]. It has particularly gained attention as a promising target for a wide range of neurological disorders. mGluR5 receptors are widely distributed and predominantly located postsynaptically in various brain regions, such as the cerebral cortex, corpus striatum, hippocampus, olfactory bulb, caudate nucleus and nucleus accumbens. Additionally, they are present in non-neuronal cells, including astrocytes and microglia [8]. These receptors have been linked to the pathogenesis of several central nervous system (CNS) disorders, including schizophrenia, addiction, anxiety, depression, Parkinson’s disease (PD), fragile X syndrome (FXS) and autism spectrum disorder (ASD) [9,10,11,12,13]. Despite their great diversity, GPCRs including mGluR are characterized by a transmembrane domain comprising 7 helical helices, an extracellular amino-terminal domain and an intracellular C-terminal domain. The endogenous ligand binding site is a globular domain consisting of two subdomains. Two conformations of this binding site have been demonstrated; (i) an open conformation that creates a large pocket between the two lobes and (ii) a closed conformation characterized by the approach of the two lobes. This folding mechanism was compared to the mechanism of action of a carnivorous plant, which gave the name of the Venus FlyTrap domain (VFT) to the glutamate binding domain [14]. In addition to this orthosteric binding site, there is an allosteric site located at the transmembrane domain. The term allosteric was first introduced by Monod et al. [15]. The need to distinguish the natural ligand binding site of a receptor from other topographically distinct binding sites has led to the use of the term orthosteric to refer to the endogenous agonist binding site.

Initial drug discovery strategies have targeted the orthosteric binding site to activate or block mGluR transmission. Recent research has convincingly shown that allosteric modulators, which interact with binding sites different from the endogenous agonist glutamate, have demonstrated superior potential. These modulators offer several advantages, such as increased subtype selectivity [16], improved blood–brain barrier penetration and the absence of desensitization that may occur with orthosteric ligands after repeated administrations [17,18]. Allosteric ligands have modulatory effects on the affinity and/or efficacy of the orthosteric agonist, an effect known as cooperativity. The intricate configuration of mGluR provides numerous opportunities for the development of allosteric modulators. Allosteric modulators can enhance the response to glutamate, known as positive allosteric modulators (PAMs), or diminish the response to glutamate, referred to as negative allosteric modulators (NAMs) [17]. mGluR5 NAMs are the most advanced category with several compounds (e.g., basimglurant, mavoglurant, dipraglurant) evaluated into clinical trials in FXS, depression or addiction [19,20,21].

### 1.2. Quantitative PET Imaging in Drug Development

Regarding these therapeutic trials, significant efforts have been dedicated to developing biomarkers and companion tests, specifically non-invasive techniques that enable imaging of glutamatergic transmission. Indeed, drug development in neuroscience is a long and costly process to advance a drug from bench to bedside. Consequently, neuroimaging can aid in enhancing decision-making processes at various stages of drug development. One very relevant quantitative effective tool is Positron Emission Tomography (PET), a noninvasive imaging modality that can be used to assess all aspects of a drug’s behavior. PET employs positron-emitter-labeled compounds to offer valuable insights into the availability of targets in both normal and pathological conditions. The labeled molecules may be drugs themselves, molecules that are substrates for a biological process, or tool compounds developed to bind with a high degree of selectivity to a specific molecular target. In addition to its contribution to the understanding of pathophysiological processes, PET imaging allows the measurement of in vivo target engagement for drug candidates with the receptor occupancy (RO) measurement. All these applications rely on the development and full characterization of specific radiotracers. No PET radiotracer that specifically targets mGluR’s orthosteric sites has been created yet. The presence of a high concentration of endogenous glutamate poses a significant challenge as it would strongly compete with the very low mass concentration of the PET radiotracer. Therefore, researchers have suggested using various radiotracers that focus on mGluR5 allosteric binding sites to study in vivo glutamate neurotransmission using PET imaging.

This review focuses on mGluR5 PET radiotracers used at several stages of CNS drug development, highlighting advances and setbacks related to the complex pharmacology of these radiotracers.

## 2. mGluR5 PET Ligands

As previously mentioned, since their discovery in 1992, mGluR5 has been implicated in pathophysiological processes leading to numerous CNS disorders. A pharmacological intervention on these receptors, and particularly blocking mGluR5, is being evaluated in various therapeutic applications. However, the great diversity observed in the pharmacology of glutamate receptors makes the development of specific drugs difficult. As such, functional imaging using PET radioligands of mGluR5 receptors is essential for (i) the study and the monitoring of disease progression, (ii) the evaluation of the therapy effect or (iii) the development of new treatments based on the quantification of receptor occupancy.

### 2.1. Ligands of the Orthosteric Site of mGluR5

The strategy for the development of mGluR5 radioligands has been performed in parallel to the research and development of candidate molecules involved in the modulation of glutamatergic transmission in clinical trials. The development of mGluR5 drugs initially focused on binding to the orthosteric site of mGluR5 [22]. Orthosteric ligands have a number of advantages such as good solubility, few interactions with brain proteins and no metabolism by cytochrome P450 (CYP450) [23]. Besides glutamate, several selective ligands of mGluR5 were discovered in the 1990s such as quisqualate, CHPG (2-chloro-5-hydroxyphenylglycine), 4CPG (4-carboxylphenylglycine) and MCPG (α-methyl-4-carboxylphenylglycine) [24,25,26,27]. Unfortunately, these ligands display a lack of selectivity with respect to the different subtypes of glutamatergic metabotropic receptors due to the fact that the orthosteric site is highly conserved across the mGlu receptor family [28]. Molck et al. performed a study evaluating orthosteric and allosteric ligand binding pockets of mGluR5. They explored the ligand recognition determinants in the orthosteric site within groups (subtype selectivity) and between groups (group selectivity) of metabotropic receptors. It has been shown that the residues less than 5 Å of the orthosteric L-glutamate binding sites of the two group I mGluRs (mGluR1 and mGluR5) are 100% conserved [29]. This has been the proof that it is impossible to develop orthosteric agonists selective for identical pockets of mGluR1/mGluR5. Moreover, the complete activation or inhibition of the receptor response obtained via orthosteric ligand binding has an additional disadvantage. Indeed, orthosteric ligands can cause many adverse effects by completely unbalancing the glutamatergic neurotransmission response [28]. Together, these reasons have largely contributed to the great difficulty in developing a radiotracer targeting the orthosteric site for in vivo imaging of mGluR5. Lastly, there are two major obstacles to the development of radioligands for the orthosteric site of mGluR5: (i) the high concentration of endogenous glutamate (on the order of millimoles after an action potential) competes with the very low mass concentration of the PET radiotracer at the orthosteric site; (ii) the development of PET tracers for a binding site for which there is a high affinity of the endogenous ligand has proven to be a difficult task.

Thus, many research groups then focused on targeting allosteric binding sites that are topographically distinct from the orthosteric site [18].

### 2.2. Ligands of the Allosteric Sites of mGluR5

Metabotropic glutamatergic receptors belonging to GPCRs are well suited for allosteric modulation due to their dynamic conformation and the diversity of potential allosteric binding pockets [29]. For mGluR5, several mutagenesis and crystallography studies [30,31] have defined an allosteric site in the transmembrane bundle (TMD). Other sites have also been proposed in the TMD domain, but their location remains unknown. The binding of a ligand to an allosteric site allows modulation of the receptor by changes in the affinity and/or efficacy of orthosteric agonists, which is called cooperativity [32]. Allosteric modulators offer advantages over orthosteric site ligands: (i) allosteric binding sites show better selectivity of mGluR subtypes than the orthosteric site [17,23]; (ii) from a therapeutic perspective, allosteric ligands reduce the risk of “hypersensitization” since they only modulate the natural response to the endogenous ligand instead of directly activating the receptor; (iii) since allosteric modulators require binding of the orthosteric ligand to have an effect, once all orthosteric sites are occupied, there can be no further allosteric effect. As a result, there is a ceiling on the biological effect known as a saturable effect.

The discovery and characterization of allosteric modulators of mGluR5 have generated considerable interest, providing new opportunities for targeted drug development for CNS disorders.

#### 2.2.1. “Cold” Ligands of the mGluR5 Allosteric Site

The discovery of allosteric modulators for mGluR5 has been particularly successful, producing diverse chemotypes spanning the entire spectrum of allosteric pharmacology (PAM, NAM, SAM). In 1999, the first selective allosteric mGluR5 antagonists were discovered (SIB-1757 and SIB-1893) [33]. Several structural modifications of these “lead” molecules led to the discovery of the compounds MPEP and MTEP, two widely used NAMs of mGluR5, whose selectivity and pharmacokinetic profile have been improved [34,35,36]. Several topographically mGluR5 allosteric sites were found [37]. By convention, the MPEP binding site is called the common binding site of mGluR5. Indeed, the majority of allosteric modulators of mGluR5 bind to the MPEP site in a highly competitive manner (example: VU0092273). In addition, partially competitive (example: VU0029251) and non-competitive modulators (example: NCFP, CPPHA, VU0357121, VU0365396) have also been found. These do not appear to bind to the common allosteric binding site MPEP, but share a functional interaction with the MPEP site [38]. Modeling studies reveal that pharmacomodulation of the molecules (e.g., to enhance metabolism) can change the pharmacology (e.g., from PAM to NAM) or even the selectivity of the subtypes (e.g., from mGluR5to mGluR3) [4]. Such a phenomenon is called a “molecular switch”. This can alter the ligand binding mode in the allosteric binding site and lead to fundamental differences between the in vitro binding and occupancy profiles of receptors in vivo [39].

#### 2.2.2. Allosteric Radioligands of mGluR5

**Figure 1:**
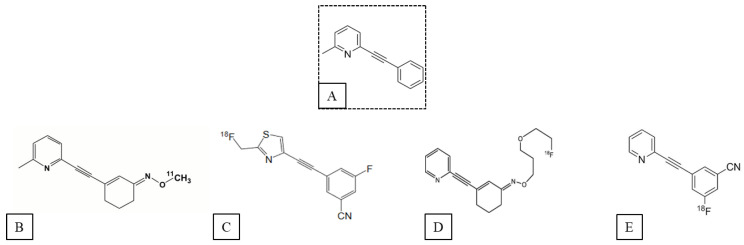
Chemical structure of (**A**) MPEP and its mGluR5 PET tracer derivatives; (**B**) ^11^C-ABP688; (**C**) ^18^F-SP203; (**D**) ^18^F-PSS232; (**E**) ^18^F-FPEB.

The majority of available allosteric radioligands of mGluR5 are based on the structure of MPEP [34] and bind to MPEP sites in a fully competitive manner. Four main compounds developed from MPEP have been described for PET imaging: [^11^C]ABP688 [40], [^18^F]FPEB [41], [^18^F]SP203 [42] and [^18^F]PSS232 [43] (Figure 1). Because [^11^C]AZD9272 was developed from a different chemotype than MPEP, its distinctive structure is of great interest [44].

[^11^C]ABP688 is a PET-negative allosteric tracer of mGluR5 that exhibits good lipophilicity (Log D = 2.4), moderate affinity (Kd between 1.7 nM [40] and 5.6 nM [45]) and rapidly reversible kinetics in the human brain. It is the most widely used PET tracer in clinical studies to assess mGluR5 receptor availability under normal, pathological or post-drug intervention conditions [46,47,48,49]. [^11^C]ABP688 is a PET tracer developed for imaging mGluR5 in humans; however, its clinical use as a PET tracer will be limited due to the need for an on-site cyclotron imposed by the short physical half-life of carbon-11. [^18^F]SP203 is another high-affinity (IC_50_ = 0.036 nM) and mGluR5-selective PET tracer (NAM) with a lipophilicity suitable for functional neuroimaging (Log D = 2.18) [42].

Compared to the previous compound, [^18^F]SP203 has the advantage of being radiolabeled with fluorine 18, whose physical half-life is 110 min instead of 20 min for carbon-11. Nevertheless, several characteristics of the tracer have limited its use in the clinic. First, [^18^F]SP203 is metabolized in vivo with the subsequent uptake of radiometabolites in bone, including the skull, which interfere with the signal in the adjacent neocortex. Second, the apparent volume of distribution of [^18^F]SP203 gradually increases during acquisition, which seems consistent with the hypothesis of radiometabolite accumulation. Indeed, the apparent volume of distribution of [^18^F]SP203 increases by approximately 10% per hour in human subjects.

To address the limitation due to the physical half-life of carbon-11, [^18^F]PSS232 is a derivative of [^11^C]-ABP688 developed by a Swiss team in 2014. It can be noted that many pharmacomodulation steps were necessary before obtaining this tracer of interest. Indeed, from ABP688, [^18^F]FE-DABP688 (Ki = 10.6 nM) and [^18^F]FPECMO (Ki = 3.6 nM) [50] are two fluorinated derivatives of ABP688 that have shown excellent properties in vitro. However, the rapid washout of both tracers in vivo in rats and the rapid defluorination of ^18^F-FPECMO quickly limited the interest of these two tracers for in vivo PET studies. To increase the lipophilicity of the previous compounds, [^18^F]PSS232, whose side chain is extended by a methylene group, was then synthesized and evaluated in vivo [51]. Within 60 min, 90% and 20% of [^18^F]PSS232 were metabolized by microsomal enzymes in rats and humans, respectively. Therefore, [^18^F]PSS232 does not allow the visualization of mGluR5 in the rat brain, due to its rapid metabolism, but could be useful for in vivo evaluation in humans. [^18^F]PSS232 is a recent radiotracer that was first injected into humans in 2018 in ten healthy male volunteers, aged 20–40 years. A period of 90 min after injection, 59.2 ± 11.1% of the total radioactivity in the plasma corresponded to an intact tracer [52].

The safety and efficacy of [^18^F]FPEB in a clinical setting were first studied in 2013 by Wong et al. [53]. Initial data on the eleven healthy volunteers indicated that the tracer was safe and well tolerated, and that the brain distribution of mGluR5 was consistent with the literature. In addition, repeatability was studied using test–retest (<10% BP_ND_) and pharmacokinetic data and indicated that [^18^F]FPEB was a suitable candidate for quantifying mGluR5 in humans in various clinical applications [54,55]. In 2017, Lohith et al. showed the superiority of [^18^F]FPEB over [^18^F]SP203 for quantifying mGluR5 in the human brain [56]. Indeed, [^18^F]SP203 generated radiometabolites that accumulate in the brain, leading to increased radioactivity uptake during the scan.

In 2012, AZD9272, a non-competitive mGluR5 antagonist that does not depend on the MPEP prototype, was developed by AstraZeneca [57]. A recent preliminary study regarding the biodistribution and dosimetry in two non-human primates suggested that [^18^F]AZD9272 is a promising radioligand to evaluate mGluR5 [58].

**Figure 2:**
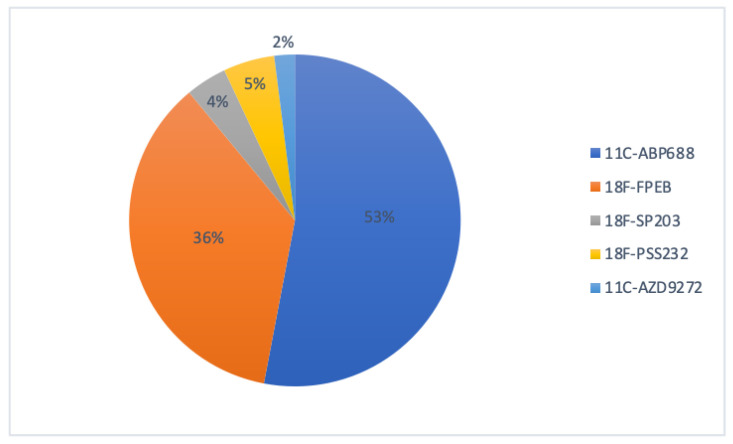
Proportional use of mGluR5 PET radioligands in clinical applications.

Among the above-mentioned mGluR5 PET radioligands, [^11^C]-APB688 has been the most widely used in clinical applications despite its carbon-11 radio-labeling. Figure 2 shows the proportion of each radiotracer used in clinical applications since 2006.

Ultimately, the complex pharmacology of mGluR5 has considerably complicated the development of its PET radioligands. Over the last 15 years, only a few negative allosteric modulators have been developed. The mGluR5 radiotracers developed satisfy strict criteria such as high specificity and selectivity for the target, low non-specific binding, high affinity and ability to cross the BBB with moderate lipophilicity. Compared with the others, [^18^F]-SP203 appears to be rapidly defluorinated, resulting in troublesome radiometabolites that lead to errors in quantification. The main pitfall of mGluR5 ligands remains as the lack of understanding of the influence of the endogenous neuromediator glutamate. Thus, sleep deprivation, circadian rhythm, smoking or age can be a major drawback in understanding the pharmacology of these tracers.

## 3. mGluR5 PET Imaging and Its Impact in CNS Drug Development

PET imaging serves as a potent and versatile technique applicable to both animal models and humans for translational research. Apart from aiding in comprehending pathophysiological processes by visualizing disease-related features, PET imaging also allows the assessment of receptor occupancy when administering pharmacological doses of specific drugs. This enables the examination of brain penetration and in vivo binding to the intended target, facilitating the correlation of these findings with plasma concentrations to predict the appropriate effective dose range for clinical investigations.

Thus, the main roles identified for CNS PET radiotracers whose target is a neuroreceptor are

(i) Imaging the pathological hallmarks of disease;
(ii) Receptor occupancies studies;
(iii) Detection of a drug’s distribution and tissue kinetics;
(iv) Monitoring treatment effect.

Preclinical or clinical studies that used the mGluR5 PET radiotracer in one of the CNS drug development stages are reported in Table 1.

## 4. Discussion

Drug development is a long and costly process, and late-stage failures result in financial costs and lost opportunities for pharmaceutical companies. The development of PET radiotracers in neuropharmacology is described and formalized by numerous articles in the literature [86]. The cornerstone in the various stages of the development of such a radiotracer is the selection of the biomedical question that the tracer should answer. Given the significant investment involved in such a process, the definition of the biomedical question must be complete and relevant. While only a few radiotracers have had a real impact in CNS pathology diagnostics ([^18^F]FDG, [^18^F]DOPA, β-amyloid ligands), today, these neuroimaging biomarkers should be considered more for their usefulness as a companion in CNS drug development. Estimating the therapeutic dose of a new CNS drug candidate is typically reliant on plasma concentration data obtained from preclinical efficacy models and human pharmacokinetics. Nonetheless, this approach frequently falls short in predicting clinical efficacy because of notable variations between species in terms of plasma protein binding, expression levels of target receptors, blood–brain barrier permeability and the drug’s affinity for the receptor. Schematically, the two main roles identified for CNS PET radiotracers whose target is a neuroreceptor are the study of receptor binding and pharmaco-imaging as a biomarker for drug development.

Despite the development and use of mGluR5 PET radioligands in the development of new drugs as described previously, several clinical trials, notably with mGluR5 NAMs, were prematurely interrupted. In an attempt to define a clinical therapeutic window for mavoglurant in FXS, [^18^F]FPEB imaging had been employed in non-human primates to measure the OR of the drug candidate [69]. Despite very promising preclinical results, and contrary to a decade of studies, Novartis announced the anticipated termination of its development program for mavoglurant, its lead mGluR5 antagonist, in 2014, following negative results in a large international clinical trial involving adults and adolescents [87]. Several hypotheses can be put forward to explain the failure of mavoglurant in clinical trials. Given the complexity of the pharmacology of mGluR and their ligands, significant attention must be paid to the interpretation of the PET signal to demonstrate changes in receptor density in vivo or the measure of the RO. To ensure meaningful in vivo RO measurement, it is essential to possess a comprehensive knowledge of the molecular pharmacological profile of both the drug being tested and the PET radiotracer and ensure that both are fully competitive.

- In the original competition model, where the endogenous ligand and the radioligand share the same binding site, the PET signal reflects the number of available receptors not occupied by the endogenous ligand. Most mGlur5 PET tracers are negative allosteric modulators (=non-competitive antagonist) that do not share the same binding site as endogenous glutamate. Thus, the theory that increasing synaptic concentrations of endogenous ligand reduces the number of receptors available for the radiotracer is challenged with an allosteric radiotracer. However, modeling studies have shown that minor changes in structure (which can occur during metabolism) can change the mode of pharmacology (e.g., from PAM to NAM or SAM or vice versa), the affinity to the allosteric site and even the selectivity to the receptor subtype. This phenomenon is called the “molecular switch” [4].
- Besides allosterism, the impact of the endogenous neuromodulator on radioligand binding can significantly limit the interpretation of various clinical PET studies and likely accounts for certain ongoing controversies within the field. Numerous preclinical and clinical studies have evaluated the influence of endogenous glutamate concentration on mGluR5 radioligand binding using a glutamatergic modulator such as N-acetylcystéine, ceftriaxone or ketamine [48,49,88,89,90,91,92,93,94]. Overall, it appears that [^11^C]ABP688 is more sensitive to changes in endogenous glutamate than the other mGluR5 radioligands. However, the precise mechanism behind this alteration in [^11^C]ABP688 binding remains unclear and cannot be attributed solely to straightforward direct competition.
- The mechanism responsible for this change in [^11^C]ABP688 binding is not clearly identified and cannot be explained by simple direct competition.
- The pharmacokinetic approach adapted to functional neuroimaging has also be discussed in a number of studies. Dynamic PET acquisitions consisting of a series of temporal images acquired over a certain time (frame) allow a precise measurement of the radiotracer kinetics. It depends on the number of receptors in the target organ, its affinity, non-specific binding, cerebral blood flow and the concentration of endogenous competitors. For the estimation of the detailed parameters (receptor density, KD, BP), the use of activity–time pharmacokinetic modeling of the tracer is required. The standard pharmacokinetic model for neuroreceptors is based on the three-compartment, two-tissue model. From the arterial blood as the first compartment, the free exchangeable radioligand in the plasma passes into the second compartment called the free compartment. The third compartment is the region of specific binding, the region of interest. The fourth compartment is a non-specific exchange compartment with the free compartment. In practice, for most radioligands, the non-specific binding compartment is in rapid equilibrium with the free compartment and the two compartments are treated as a single compartment. Yet, there is great heterogeneity in the pharmacokinetic models used and there is no consensus on the reference region to quantify the non-specific binding.
- Finally, the concept of receptor internalization by endogenous agonist stimulation is now well described for GPCR. But very little data are available on the ability of an allosteric radioligand to bind to its transmembrane allosteric binding site when mGluR5 is internalized.

Given the failure of clinical trials of mGluR5 modulators and the observed variability in subcortical radioligand uptake in PET studies with FXS subjects, it may be suggested that the future of mGluR5 PET radioligands lies in the potential to identify individual subjects with lower regional uptake of the radioligand. This could then be used to objectively select subjects for FXS treatment trials targeting the mGluR5 pathway. Furthermore, as the balance between glutamate and gamma-aminobutyric acid (GABA) is of paramount importance in the brain, playing a fundamental role in maintaining proper brain function and overall neuronal activity, PET studies combining radiotracers could be envisaged. Combining different radiotracers in a PET study is a powerful and versatile approach that provides a more comprehensive and integrated view of the biological processes under investigation.

For mGluR5 PET exploration, [^11^C]ABP688 and [^18^F]FPEB appear to be the most promising. [^18^F]FPEB has a longer half-life (110 min) due to the longer half-life of fluorine-18, which allows for more extended imaging sessions and the potential for shipping to distant PET centers. It is suitable for multicenter studies. In contrast, it has a lower specific signal-to-noise ratio compared to [^11^C]ABP688 due to higher non-specific binding, which can make quantification more challenging in some cases. Ultimately, the choice between [^18^F]FPEB and [^11^C]ABP688 will depend on factors such as the specific research question, the availability of a cyclotron for on-site [^11^C]ABP688 production, the desired imaging duration and the need for multicenter studies.

## 5. Conclusions

mGluR5 has garnered significant interest as a crucial target for pharmaceutical companies, mainly due to its wide-ranging functions and documented involvement in CNS disorders. Particularly, the therapeutic potential of its modulators has drawn attention. Allosteric modulators, with their enhanced selectivity and control over disease-related receptors, may offer a competitive edge over conventional drugs. mGluR5 is an interesting target for understanding the pathophysiology of CNS disorders and drug development, but also for neuroimaging. There is a real need for relevant biomarkers of CNS pathologies and PET imaging is an attractive molecular imaging modality that can provide quantitative measurements such as receptor concentrations. Whether biochemical, genetic or imaging, a biomarker must be relevant, sensitive, specific and answer the biomedical question posed with high accuracy. However, given the complexity of GPCR signaling and the multitude of factors that may influence the interpretation of the PET signal (pharmacology of the ligand/receptor complex, pharmacokinetic modeling, etc.), and as demonstrated by the heterogeneity of the results of the various studies, it is critical to rigorously evaluate the interaction between each PET ligand and the drug lead or drug candidate being investigated.

## Figures and Tables

**Table 1:** Studies with an mGluR5 PET radiotracer in one of the CNS drug development stages.

Radioligand	PET Application in Drug Development	Drug/Disease	Population	Main Findings	Reference
[^11^C]ABP688	Hallmark of disease	Major depressive disorder	Clinical: 11 un-medicated individuals with MDD and 11 matched healthy comparison subjects	Lower levels of regional mGluR5 binding in the prefrontal cortex, the cingulate cortex, the insula, the thalamus and the hippocampus in the depression group relative to the comparison group.	(2011) [59]
	RO study	AZD2066	Clinical: 6 healthy volunteers after different doses of AZD2066	AZD2066 displaced [^11^C]ABP688 from mGluR5 binding sites in the human brain. The estimated Ki was around 1200 nM, suggesting that approximately 50% occupancy was achieved at Cmax with the highest dose (13.5 mg).	(2013) [60]
	RO study	Fenobam	Preclinical: 4 baboons’ PET at baseline condition vs. after intravenous treatment with fenobam at different dose levels (0.3–1.33 mg/kg)	In vivo binding of [^11^C]ABP688 was blocked by pre-treatment with fenobam in a dose-dependent, saturable manner, approaching close to full occupancy (>90%) at a dose of 1.33 mg/kg.	(2014) [61]
	Hallmark of disease	Major depressive disorder	Clinical: 20 elderly (mean age: 63.0 ± 6.3) subjects with MDD and 22 healthy volunteers in the same age range	No significant difference in [^11^C]ABP688 binding was observed between elderly subjects with MDD and healthy volunteers.	(2015) [62]
	Hallmark of disease	Behavioral variant frontotemporal dementia	Clinical: 5 bvFTD patients and 10 healthy volunteers	BvFTD patients showed widespread decrements in [^11^C]ABP688 BP_ND_ throughout frontal, temporal and subcortical areas.	(2016) [63]
	Hallmark of disease	Schizophrenia	Clinical: 15 individuals with schizophrenia and 15 healthy controls.	Distribution volume ratio in the 15 individuals with schizophrenia did not differ from that of the 15 controls.	(2017) [46]
	Hallmark of disease	Huntington’s disease	Preclinical: 18 heterozygous mice (Q175 Mouse Model of Huntington’s Disease) and 18 wild-type (WT) at 3 different time points (6, 9 and 13 months old)	Reduction in [^11^C]ABP688 binding in the striatum and cortex of heterozygous mice, compared with WT mice, as well as a temporal decline.	(2018) [64]
	Hallmark of disease	Alzheimer’s disease	Clinical: 9 subjects with AD and 10 cognitively healthy controls	Reduction in mGluR5 binding in the hippocampus and amygdala in AD group.	(2020) [65]
	Drug distribution and RO study	Mavoglurant	Clinical: 6 subjects divided into 2 cohorts at different doses (25,100, 200, 400 mg) of mavoglurant and different periods.	Mavoglurant passes the blood–brain barrier and induces a dose/exposure-dependent displacement of [^11^C]ABP688 bound to mGlu5 receptors in humans in vivo. A single oral dose of 400 mg induced an estimated displacement of 63% at a scan time of 3–4 h post dose, inferring a receptor occupancy estimate of nearly 85%.	(2021) [66]
	Hallmark of disease	Major depressive disorder	Clinical: 20 non-smoking MDD patients and 18 matched non-smoking healthy controls.	Significant differences in frontal mGluR5 availability depending on the level of social avoidance in drug-naïve non-smoking MDD patients.	(2022) [67]
[^18^F]PSS232	Hallmark of disease	Neuroinflammation model	Preclinical: 4 LPS-induced animal models of neuroinflammation and 4 control mice	LPS-induced neuroinflammation increased mGluR5 levels in mouse brain.	(2019) [68]
[^18^F]FPEB	RO study	Mavoglurant	Preclinical: 2 male cynomolgus monkeys PET at baseline condition vs. after intravenous treatment with mavoglurant at 2 different doses (0.074 and 0.34 mg/kg)	Measured RO for mavoglurant was 73% at the 0.34 mg/kg dose and 51% at 0.074 mg/kg. The current data would predict that ≥80% RO is required for efficacy.	(2012) [69]
	RO study	-VU0409106 (NAM) -VU0092273 (ago-PAM) -VU0360172 (PAM)	Preclinical: 8, 5 and 7 rats, respectively, after IP injection of increasing doses of treatment (3–100 mg/kg)	VU0409106: ED_50_ = 7.5 mg/kg VU0092273: ED_50_ = 17.8 mg/kg VU0360172 does not significantly displace [^18^F]FPEB binding to mGlu5 in vivo, demonstrating that RO does not predict in vivo efficacy for this mGlu5 PAM.	(2015) [39]
	Hallmark of disease	Amyotrophic lateral sclerosis	Preclinical: 4 ALS mice expressing SOD1-G93A gene and 4 control base mice (C57/BL6)	In the whole brain, the binding potential increased by 49 ± 9% from base mice to ALS-type mice and further enhanced by 23 ± 4% during disease progression.	(2015) [70]
	Hallmark of disease	Major depressive disorder	Clinical: 30 MDD and 35 HC	No significant between-group differences were observed in mGluR5 VT or DVR	(2017) [71]
	Hallmark of disease	Autism spectrum disorder	Clinical: 6 ASD patients and 3 control subjects	Significantly higher [^18^F]FPEB binding potential in the postcentral gyrus and cerebellum of individuals with autism	(2018) [72]
	Hallmark of disease	Alcohol Dependence	Clinical: 16 recently abstinent alcohol-dependent subjects and 32 age-matched controls	mGluR5 availability was lower mainly in limbic regions of alcohol-dependent subjects than in controls, ranging from 14% in the posterior cingulate cortex to 36% in the caudate nucleus.	(2018) [73]
	Hallmark of disease	Alzheimer disease	Preclinical: 4 10-month-old male 5xFAD transgenic mice models and 4 10-month-old wild type (WT) mice were used as control	mGluR5 in the hippocampus and the striatum was significantly lower in 5xFAD mice compared to control animals.	(2018) [74]
	Hallmark of disease	Cocaine addiction	Preclinical: 42 rats before and after sucrose or intravenous cocaine self-administration, during withdrawal and during relapse.	Only cocaine self-administration induced a decrease in [^18^F]FPEB binding	(2018) [75]
	Hallmark of disease	Parkinson’s disease	Clinical: 9 patients with PD and 8 healthy volunteers (HV)	[^18^F]FPEB BP_ND_ values were slightly more than 20% higher in PD than HVs in several mesocortical regions, including the bilateral putamen, hippocampus and amygdala.	(2018) [76]
	Hallmark of disease	Autism spectrum disorder	Preclinical: 6 Shank3B−/− mice and 6 control mice	Shank3B−/− mice showed significantly increased BP_ND_ compared to the control mice in the hippocampus, thalamus, striatum and amygdala	(2019) [77]
	Hallmark of disease	Alzheimer disease	Clinical: 16 individuals with amnestic mild cognitive impairment (MCI) due to AD or mild AD dementia who were positive for brain amyloid were compared to 15 cognitively normal (CN) participants who were negative for brain amyloid.	Significant reduction (43%) in mGluR5 binding in the hippocampus of AD compared to participants.	(2020) [78]
	Monitoring treatment effect	Ebselen	Preclinical: Dawley rats were randomized to receive either ebselen (5 mg/kg, *n* = 4) or vehicle (*n* = 4).	Acute administration of ebselen potentially decreases synaptic glutamate levels, as measured by an increased brain uptake of [^18^F]FPEB	(2020) [79]
	Hallmark of disease	Fragile X Syndrome	Clinical: 9 men with FXS and 8 with typical development (TD)	mGluR5 expression was significantly reduced in cortical and subcortical regions of men with FXS in contrast to age-matched men with TD.	(2020) [80]
	Hallmark of disease	Autism spectrum disorder and Fragile X Syndrome	Clinical: 10 men with FXS, 7 with ASD and 19 with typical development (TD)	In contrast to participants with TD, mGluR5 expression was significantly increased in the cortical regions of participants with IASD and significantly reduced in all regions of men with FXS.	(2021) [81]
	Hallmark of disease	Fragile X Syndrome	Clinical: 8 males with FXS and 8 age- and gender-matched controls	Patients with FXS showed lower [^18^F]FPEB binding potential, reflecting reduced mGluR5 availability, than the healthy controls throughout the brain, with significant group differences in insula, anterior cingulate, parahippocampal, inferior temporal and olfactory cortices.	(2021) [82]
	Hallmark of disease	Post-traumatic stress disorder and major depressive disorder	Clinical: 28 PTSD, 21 MDD and 28 healthy adults were matched for age, gender and smoking status.	Significant relationship between frontolimbic mGluR5 availability and performance on tests of attention in individuals with MDD and PTSD	(2022) [83]
	Hallmark of disease	Bipolar disorder and major depressive disorder	Clinical: Individuals with BD (*n* = 17 depressed; *n* = 10 euthymic), MDD (*n* = 17) and healthy control (HC) individuals (*n* = 18)	mGluR5 was lower in BD versus MDD and HC groups, with no difference between MDD and HC groups.	(2022) [84]
	Hallmark of disease	Autism spectrum disorder	Clinical: 12 adult males with ASD and 14 healthy adult males	mGluR5 binding was significantly increased in the brain of ASD vs. controls groups	(2023) [85]

MDD, Major depressive disorder; RO, Receptor occupancy; BP_ND,_ non-displaceable binding potential; ALS, Amyotrophic lateral sclerosis; VT, Volume of Distribution; DVR, Distribution Volume Ratio; ASD, Autism Spectrum Disorder; PD, Parkinson’s disease; HV, Healthy volunteers; HC, Healthy control FXS, Fragile X Syndrome; PTSD, Post-traumatic stress disorder; BD, Bipolar disorder; WT, Wild Type; IP, Intraperitoneal; CN, Cognitively normal; TD, Typical development; bvFTD, Behavioral variant frontotemporal dementia.

## Data Availability

Data sharing not applicable.
